# Giant pelvic angiomyofibroblastoma: case report and literature review

**DOI:** 10.1186/1746-1596-9-106

**Published:** 2014-06-03

**Authors:** Ping Qiu, Zhe Wang, Yao Li, Guangbin Cui

**Affiliations:** 1 Department of Endocrinology, The First Affiliated Hospital of Chengdu Medical College, Chengdu, Sichuan 610500, People’s Republic of China; 2 Department of Pathology, State Key Laboratory of Cancer Biology, Xijing Hospital and School of Basic Medicine, Fourth Military Medical University, Xi’an, Shanxi 710032, People’s Republic of China; 3 Department of Radiology, Tangdu Hospital, Fourth Military Medical University, Xi’an, Shanxi 710038, People’s Republic of China

**Keywords:** Angiomyofibroblastoma (AMF), Aggressive angiomyxoma (AAM), Pelvis

## Abstract

Angiomyofibroblastoma (AMF) is a rare, benign, soft-tissue tumor, which predominantly occurs in the vulvovaginal region of middle-aged women. It is clinically important to distinguish an AMF from other stromal cell lesions. Here, we report the case of a 32-year-old woman with a rare, giant pelvic AMF, which showed a benign clinical course. The tumor was located in the cul-de-sac of Douglas. It was well demarcated, hypocellular, edematous and composed of spindle-shaped and oval stromal cells aggregating around thin-walled blood vessels. The tumor cells had abundant eosinophilic cytoplasm, and expressed estrogen receptors, progesterone receptors and desmin. Mitotic figures were absent. It is important to distinguish AMFs from aggressive angiomyxomas because both occur at similar sites but show different clinical behaviors. Most AMFs and aggressive angiomyxomas have the same immunohistochemical phenotype. The well-circumscribed borders of AMF are the most important characteristic that distinguish it from aggressive angiomyxomas. AMFs rarely recur after complete surgical excision.

**Virtual Slides:**

The virtual slide(s) for this article can be found here: http://www.diagnosticpathology.diagnomx.eu/vs/5510813471244189.

## Background

Angiomyofibroblastoma (AMF) is a rare soft-tissue neoplasm that most frequently affects the lower genital tracts of young to middle-aged women. This tumor belongs to the group of genital mesenchymal tumors [1]. These tumors commonly involve the vulva, perineum, vagina, uterine cervix [2-4] and the inguinoscrotal regions of men [5], but only rarely occur in the pelvis or retroperitoneum. Rare recurrences of AMF after excision have been reported in the literature [6].Here, we report a case of a giant AMF in an uncommon location, namely, the pelvic region. We also emphasize the difficulty in making an accurate preoperative diagnosis. The differential diagnosis for such a tumor includes an aggressive angiomyxoma (AAM), which unlike AMF, does not have well-defined margins.

## Case presentation

### Clinical history

**Figure 1:**
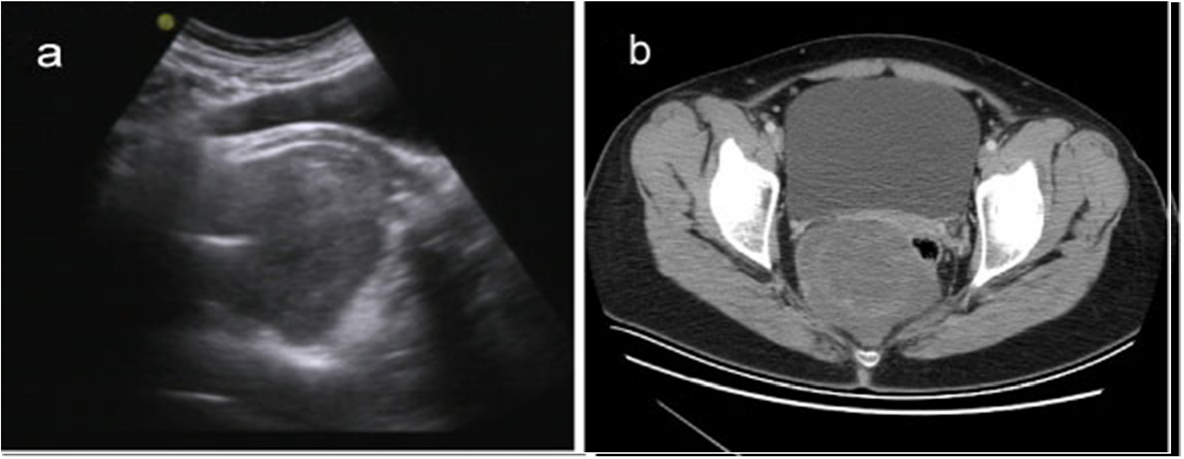
**Image feature of tumor pre-operation. (a)** Transvaginal ultrasonography shows an oval, moderately echoic mass in close proximity to the cul-de-sac of Douglas. **(b)** Pelvic CT scan shows a well-circumscribed tumor of homogeneous intensity located anteroinferior to the sacrum.

A 32-year-old woman presented with intermittent dysuria for 1 month and an exacerbation of this symptom 2 weeks ago. An ultrasound examination revealed a pelvic mass, and she was admitted to our hospital for further investigation and treatment. Her medical history was unremarkable. Her menstrual cycle was regular, and she did not have dysmenorrhea or menorrhagia. Her family history was non-significant. The patient is a farmer and she was not exposed to any environmental hazards. A physical examination did not reveal any abnormalities. Transvaginal ultrasonography showed an oval, moderately echoic mass in close proximity to the cul-de-sac (pouch) of Douglas. The tumor had a thick capsule, was well demarcated and measured 13.2 × 5.8 × 7.8 cm. A small, hypoechoic area was observed within the mass during the ultrasound examination (Figure 1a). Computed tomography (CT) showed that the tumor was located anteroinferior to the sacrum within the pelvic cavity (Figure 1b). It was well circumscribed and showed fiber-like signals of homogeneous intensity. The tumor was moderately enhanced on contrast-enhanced CT. The rectum and uterus were compressed toward the left and anterior directions. No enlarged lymph nodes were seen in the pelvic cavity. The patient underwent an exploratory laparotomy. A soft, regular-shaped tumor was found behind the rectum, which was pushed to the left. The tumor had an intact capsule and did not adhere to or invade the peripheral tissues. Then complete local excision was carried out. The patient’s postoperative course has been uneventful after 1 year of follow-up.

### Pathological findings

**Figure 2:**
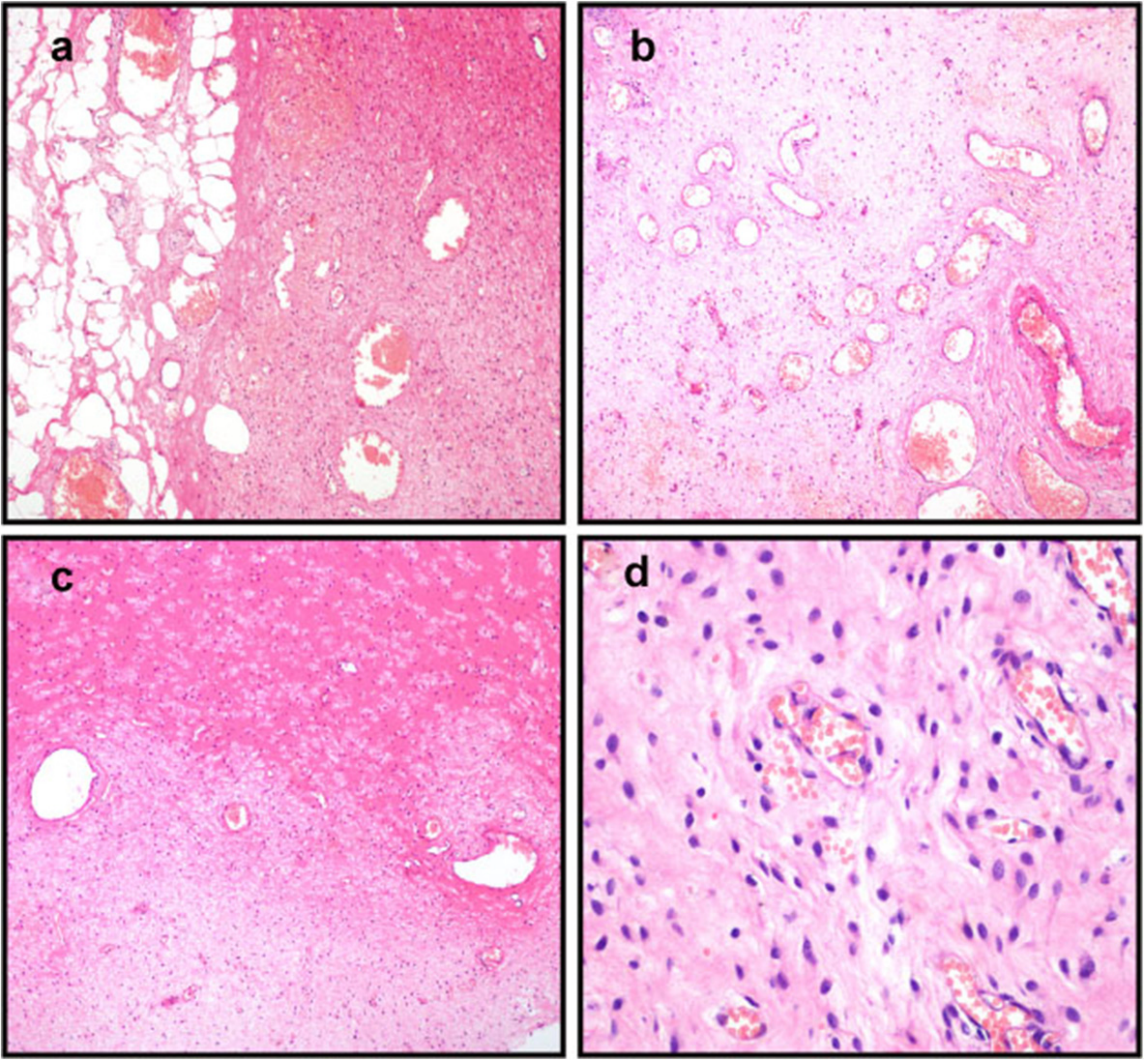
**Histopathological examination of tumor post-operation. (a)** The tumor is well demarcated from the surrounding fat tissues. **(b)** Abundant thin-walled blood vessels can be seen in the tumor. **(c)** The stroma of the tumor is hyalinized or edematous, and appears hypocellular in some areas. **(d)** The tumor is composed of bland, plump, spindle-shaped or oval cells that are frequently aggregated around thin-walled blood vessels (H&E: 100 x).

**Figure 3:**
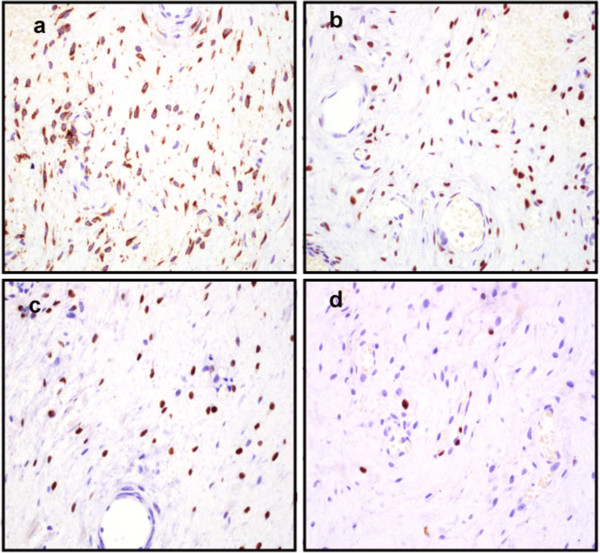
**Immunostaining of the tumor cells. (a)** The tumor cells were positive for desmin. **(b)** The tumor cells were immunoreactive with estrogen receptor **(c)** and progesterone receptor. **(d)** The tumor cells showed a very low Ki-67 index (IHC: 100 x).

The excised tumor was well circumscribed and measured 10 × 6 × 5 cm. The cut surface appeared tan in color and homogeneous with large flesh-like tissue. No hemorrhage, necrosis or cystic changes were observed. Under a microscope, the tumor appeared well demarcated from the surrounding fat tissue (Figure 2a), and was characterized by alternating hypercellular and hypocellular edematous zones in which abundant thin-walled blood vessels were haphazardly distributed (Figure 2b). The stroma of the tumor appeared hyalinized or edematous and was hypocellular in some areas (Figure 2c). Interstitial myxoid degeneration was also seen in the lesion. The tumor cells were spindle-shaped or stellate, with fine chromatin and inconspicuous nucleoli (Figure 2d). Mitotic figures were absent. Bland-looking myoid tumor cells were scattered in the fibromxyoid stroma, and sometimes aggregated around the blood vessels. Immunohistochemistry (IHC) revealed that the tumor cells were positive for desmin (Figure 3a), estrogen receptor (Figure 3b), progesterone receptor (Figure 3c) and vimentin; the Ki-67 proliferation index was less than 5% (Figure 3d). The tumor was negative for smooth muscle actin, S-100 protein, CD34, CD117 and β-catenin. These distinctive pathological and IHC features indicated a diagnosis of AMF.

## Discussion

AMF is a rare, benign, soft-tissue tumor that shows myofibroblastic differentiation and represents neoplastic proliferation of stromal cells. AMF was first described by Fletcher et al. in 1992 [1]. AMF is composed of myofibroblastic cells and prominent thin-walled blood vessels within a fibromxyoid stroma. Its cause and pathogenesis are unknown at present. Since AMF is rare, no statistical data are available about its incidence in the general population. In most reports, AMF has occurred in women aged between 20 and 50 years, i.e., during the reproductive years [1,2]. There are very rare reports of AMF occurring in male patients [5,7]. Most cases of AMF are benign, and only one case with sarcomatous transformation has ever been reported [8]. A rare lipomatous variant of AMF with local invasion has also been reported [9,10].

In most patients, AMF presents as a painless neoplasm located in the superficial regions of the lower female genital tract, such as the vulva and vagina [4], and are therefore easily detected. Tumors arising in the cervix, uteri or urethral region sometimes present with obstructive symptoms [11]. Tumors in the pelvis, iliac fossa or peritoneal cavity are extremely rare, and usually grow insidiously and reach a massive size before they are detected [12-15]. Their greatest dimensions have ranged from 3.8 to 25 cm [12-15]. To our knowledge, only four cases of pelvic AMF have been reported at least so far (Table 1). Table 1 summarizes the major clinical and pathological features of previously reported cases. Similar to AMFs arising in other sites, the pelvic AMFs were well demarcated.

**Table 1:** Clinical features of female pelvic AMF

**References**	**Age**	**Site**	**Size(cm)**	**Duration**
Lim, et al. [14].	48	Posterior perivesical space	3.8 × 3.5 × 2.8	Not mentioned
Quintero, et al. [12].	28	Pelvic retroperitoneum	12 × 9.6 × 8	1 year
Kobayashi, et al. [15].	28	Pelvic cavity	25 × 14 × 4	2 years
Menendez, et al. [13].	49	Ischiorectal fossa	5.5 × 4.3 × 2	2 years

Several reports have analyzed the imaging features of AMFs. On perineal ultrasonography, AMFs appear as a soft-tissue mass with inhomogeneous mixed echogenicity, which corresponds to the cellular inhomogeneity found on histopathological examination. Therefore, the ultrasonographic characteristics of AMFs may help to differentiate them from other mesenchymal neoplasms [16]. On CT imaging, AMFs most likely show moderate-to-strong enhancement, which may reflect the prominent vascularity of these tumors [16]. There have been three cases of AMF which showed well-defined margins and heterogeneous or homogeneous intermediate signal intensity on CT. In the current case, the small, hypoechoic area inside the mass observed on ultrasound examination may be attributable to the flesh-like structures seen on gross examination.

IHC showed that the tumor expressed estrogen and progesterone receptors, which suggests that it might have originated as a neoplastic proliferation of hormonally responsive mesenchymal cells.

As AMF has a benign clinical course, it should be differentiated from other tumor-like lesions of the vulvovaginal region including Bartholin cysts,benign lipoma,fibroepithelial stromal polyps and cellular angiofibromas[1-4,9,10].

In the present case, AMF could be readily distinguished from Bartholin cysts and fibroepithelial stromal polyps according to histopathological and immunohistochemical findings. The distinction between AMF and cellular angiofibroma, both of which have a characteristic vascular network and spindle cell component, is relatively subtle. However, cellular angiofibroma is characterized by the presence of spindle cell lipomas and thick-walled vessels. Meanwhile, cellular angiofibromas lack ER and PR [9].

Aggressive angiomyxoma(AAM) is most likely to be confused with AMF because it shares many features with AMF including age at presentation, location, clinical manifestations and pathological entities. However, AAM presents as a malignant, locally infiltrative, non-metastasizing stromal neoplasm with a strong tendency to recur. On microscopic examination, AMFs generally show much higher cellularity, more numerous blood vessels and more frequent plump or short spindle-shaped cells; in contrast, AAM cells are sparsely and diffusely distributed, without the characteristics of alternating density and aggregation around small blood vessels. AAMs show more distinctive myxoid degeneration than AMFs [1,17]. AMFs are characterized by the expression of vimentin, desmin and CD34, suggesting an undifferentiated mesenchymal tumor with preferential myofibroblastic differentiation. Desmin expression was previously thought to be specific for AMFs, but positive expression of desmin has been found in some cases of AAMs. Most AAMs are also positive for estrogen and progesterone receptors. Hence, desmin, estrogen receptor and progesterone receptor are no longer considered reliable markers for distinguishing AAMs from AMFs [17]. After surgical treatment, 30% of AAMs have been found to relapse within 2 years [1]. Since the morphology and IHC markers of AMF are similar to those of AAM, differentiation between these two tumors is largely based on the appearance of the tumor margins (AMFs are well demarcated).

As AMF is a commom perineum-site-specific stromal tumour, a benign neoplasm of dendritic fibromyxolipoma (DFML) in rare sites such as perineum regions should be differentiated from AMF. Zhang XJ et al. [18] recently reported the case of a woman of similar age to our current case with a slow-growing, painless, subcutaneous tumor in the right inguinal and perineal regions which was diagnosed as DFML lately. Histologically, DFML is mainly composed of a proliferation of small spindle or stellate cells that are variably admixed with mature adipose tissue embedded within an abundant myxoid and collagenized stroma. Immunohistochemically, the spindle and stellate cells are strongly positive for vimentin, CD34, and bcl-2 antibodies but not for smooth muscle actin and desmin. The above-mentioned histopathological and immunohistochemical findings can easily distinguish AMF from DFML.

The pathological differential diagnosis should also include inflammatory myofibroblastic (IMT) which has myofibroblastic differentiation [19]. These are rare benign lesions that are predominately located in the lung. Microscopic examination reveals a proliferation of regular myofibroblastic spindle cells arranged within a fibrous, myxoid or calcified stroma, associated with an inflammatory component of lymphocytes and plasmacytes but without blood vessels [19]. Immunohistochemical studies show reactivity for vimentin, smooth muscle actin and ALK.

The treatment of choice for AMF is simple total excision, which is usually curative, and there are almost no incidences of recurrences or metastasis after complete excision, which confirms the benign nature of AMF [20]. AMF shows no propensity for infiltrative growth.

## Conclusion

Pelvic AMF is extremely rare but is benign. Its preoperative diagnosis and differentiation from other soft-tissue tumors are challenging. The combination of radiological data, and histological and IHC findings can confirm the diagnosis.

## Consent

Written informed consent was obtained from the patient to approve the publication of this Case Report and any accompanying images.

## Competing interests

The authors declare that they have no competing interests.

## Authors’ contributions

PQ drafted the manuscript and performed the literature review. YL was responsible for collecting the patient material. ZW conducted the analysis of the histological documentation and corrected the manuscript.GBC performed image features. All authors read and approved the final manuscript.

## References

[B1] Fletcher CD Tsang WY Fisher C Lee KC Chan JK Angiomyofibroblastoma of the vulva. A benign neoplasm distinct from aggressive angiomyxoma Am J Surg Pathol 1992 16 373 382 10.1097/00000478-199204000-00006 1314521

[B2] Babala P Biro C Klacko M Miklos P Ondrus D Angiomyofibroblastoma of the cervix uteri: a case report Klin Onkol 2011 24 133 136 21638997

[B3] Mortele KJ Lauwers GJ Mergo PJ Ros PR Perineal angiomyofibroblastoma: CT and MR findings with pathologic correlation J Comput Assist Tomogr 1999 23 687 689 10.1097/00004728-199909000-00007 10524846

[B4] Naheed S Upadhyay K Pradeep K Angiomyofibroblastoma of the vulva J Obstet Gynaecol 2011 31 554 555 10.3109/01443615.2011.581314 21823872

[B5] Lee SH Yang JW Do JM Seo DH Jung JH Chung KH Lee JS Hyun JS Angiomyofibroblastoma-like tumor of the scrotum Korean J Urol 2010 51 365 367 10.4111/kju.2010.51.5.365 20495703 PMC2873894

[B6] Saleh MM Yassin AH Zaklama MS Recurrent angiomyofibroblastoma of the vagina: a case report Eur J Gynaecol Oncol 2007 28 324 17713105

[B7] Ding G Yu Y Jin M Xu J Zhang Z Angiomyofibroblastoma-like tumor of the scrotum: a case report and literature review Oncol Lett 2014 7 435 438 24396463 10.3892/ol.2013.1741 PMC3881198

[B8] Nielsen GP Young RH Dickersin GR Rosenberg AE Angiomyofibroblastoma of the vulva with sarcomatous transformation (“angiomyofibrosarcoma”) Am J Surg Pathol 1997 21 1104 1108 10.1097/00000478-199709000-00016 9298888

[B9] Laskin WB Fetsch JF Tavassoli FA Angiomyofibroblastoma of the female genital tract: analysis of 17 cases including a lipomatous variant Hum Pathol 1997 28 1046 1055 10.1016/S0046-8177(97)90058-7 9308729

[B10] Vora S Gaba ND Stamatakos MD Lipomatous angiomyofibroblastoma: a case report of a unique vulvar mass J Reprod Med 2011 56 347 350 21838167

[B11] Kitamura H Miyao N Sato Y Matsukawa M Tsukamoto T Sato T Angiomyofibroblastoma of the female urethra Int J Urol 1999 6 268 270 10.1046/j.1442-2042.1999.00059.x 10375192

[B12] Quintero C Sasken H Houck KL Hernandez E Angiomyofibroblastoma of the retroperitoneum: a case report J Reprod Med 2007 52 741 744 17879839

[B13] Menendez SP Villarejo CP Padilla VD Munoz AV Gonzalez LL Martin FJ Angiomyofibroblastoma of the right ischiorectal fosa Cir Cir 2010 78 448 450 21219818

[B14] Lim KJ Moon JH Yoon DY Cha JH Lee IJ Min SJ Angiomyofibroblastoma arising from the posterior perivesical space: a case report with MR findings Korean J Radiol 2008 9 382 385 10.3348/kjr.2008.9.4.382 18682679 PMC2627271

[B15] Kobayashi T Suzuki K Arai T Sugimura H Angiomyofibroblastoma arising from the fallopian tube Obstet Gynecol 1999 94 833 834 10.1016/S0029-7844(99)00362-2 10546747

[B16] Kim SW Lee JH Han JK Jeon S Angiomyofibroblastoma of the vulva: sonographic and computed tomographic findings with pathologic correlation J Ultrasound Med 2009 28 1417 1420 19778894 10.7863/jum.2009.28.10.1417

[B17] Steeper TA Rosai J Aggressive angiomyxoma of the female pelvis and perineum. Report of nine cases of a distinctive type of gynecologic soft-tissue neoplasm Am J Surg Pathol 1983 7 463 475 10.1097/00000478-198307000-00009 6684403

[B18] Zhang XJ Zhou S Nie K Chen DF Kui GJ Zhang XH Dendritic fibromyxolipoma in the right inguinal and perineum regions: a case report and review of the literature Diagn Pathol 2013 8 157 163 10.1186/1746-1596-8-157 24053125 PMC4015604

[B19] Nawal H Laila C Mohammed R Meryem B Sara B Youssef B Siham T Mustapha H Afaf A A rare tumor of the lung: inflammatory myofibroblastic tumor Diagn Pathol 2012 7 83 86 10.1186/1746-1596-7-83 22805416 PMC3482609

[B20] Stewart C Angiomyofibroblastoma of the vagina Pathology 2009 41 199 200 10.1080/00313020802579383 19152197

